# Controlled elevated temperatures during early-mid gestation cause placental insufficiency and implications for fetal growth in pregnant pigs

**DOI:** 10.1038/s41598-020-77647-1

**Published:** 2020-11-26

**Authors:** Weicheng Zhao, Fan Liu, Alan W. Bell, Hieu H. Le, Jeremy J. Cottrell, Brian J. Leury, Mark P. Green, Frank R. Dunshea

**Affiliations:** 1 Faculty of Veterinary and Agricultural Sciences, University of Melbourne, Parkville, 3010 Australia; 2 Rivalea Australia Pty Ltd, Corowa, 2646 Australia; 3 Department of Animal Science, Cornell University, Ithaca, 14853-4801 USA; 4 School of BioSciences, University of Melbourne, Parkville, 3010 Australia; 5 Faculty of Biological Sciences, The University of Leeds, Leeds, LS2 9JT United Kingdom

**Keywords:** Reproductive biology, Animal physiology

## Abstract

It is known that pig offspring born from pregnant pigs exposed to elevated ambient temperatures during gestation have altered phenotypes, possibly due to placental insufficiency and impaired fetal growth. Therefore, the objective of this study was to quantify the effect of maternal heat exposure during early-mid gestation, when pig placentae grow heavily, on placental and fetal development. Fifteen pregnant pigs were allocated to thermoneutral (TN; 20 °C; n = 7) or cyclic elevated temperature conditions (ET; 28 to 33 °C; n = 8) from d40 to d60 of gestation. Following euthanasia of the pigs on d60, placental and fetal morphometry and biochemistry were measured. Compared to TN fetuses, ET fetuses had increased (P = 0.041) placental weights and a lower (P = 0.013) placental efficiency (fetal/placental weight), although fetal weights were not significantly different. Fetuses from ET pigs had reduced (P = 0.032) *M. longissimus* fibre number density and a thicker (P = 0.017) placental epithelial layer compared to their TN counterparts. Elevated temperatures decreased (P = 0.026) placental mRNA expression of a glucose transporter (GLUT-3) and increased (P = 0.037) placental *IGF-2* mRNA expression. In conclusion, controlled elevated temperatures between d40 to d60 of gestation reduced pig placental efficiency, resulting in compensatory growth of the placentae to maintain fetal development. Placental insufficiency during early-mid gestation may have implications for fetal development, possibly causing a long-term phenotypic change of the progeny.

## Introduction

Global warming is driving an increased frequency and intensity of summer heatwaves, especially in countries such as Australia^1,2^. Consequently, the livestock industries must adapt an increased incidence of extreme summer heat events in the future^3,4^. Due in part to their large size and low heat tolerance, domestic pregnant pigs are susceptible to high ambient temperatures, which are a contributing factor to reduced reproductive efficiency over the summer months^5–7^. Colloquially referred to as summer infertility, it causes significant economic burden to the industry^8^, and this unprecedented challenge is expected to continue as the impacts of climate changes increasingly assert themselves^2^.

Summer infertility is a multi-faceted syndrome, with effects on both the pregnant pigs and their progeny^9^. This includes delayed puberty^7^, prolonged weaning to oestrus intervals^5^ and reduced conception rates^10^, possibly due to compromised ovarian function and follicle quality^11,12^. Notably, it has been demonstrated that exposing pregnant pigs to high environmental temperatures during gestation reduces litter size^13,14^, piglet birthweights^15–17^, and the number of piglets born alive^15^. It is known that pigs reared under elevated environmental temperatures are more prone to deposit adipose tissue than pigs reared under optimal ambient conditions^18,19^. This has also been observed in progeny born from pregnant pigs that experienced heat stress during gestation, even when they were reared under normal postnatal environmental conditions^16,20^. These findings indicate that elevated temperatures in utero may cause a long-term postnatal change in phenotypes of the progeny.

An important mechanism by which phenotypes of the progeny are affected is via placental programming^21,22^. Placental efficiency, known as the ratio between fetal and placental weights at a given pregnancy stage, is a measure of the influence of placental function on pregnancy outcomes^23^. There are many factors influencing placental efficiency in farm animals, including (1) nutrient transporter capacity and abundance on the surface membrane of placentae^24,25^, (2) physical morphology of placentae, such as placental size and the thickness of the maternal–fetal surface^26^, and (3) placental angiogenesis^27,28^, leading to altered uterine and umbilical blood flows^29,30^.

Morphologically, growth restriction in placental and fetal development due to maternal heat stress has been observed in pregnant sheep^31–34^. Notably, the impact of maternal heat exposure during gestation on placental and fetal development in pigs remains poorly understood and merits further research. As pig placentae develop rapidly in weight, length and surface area during the first half of gestation^35^, it is likely that this is a key developmental period that may be sensitive to high environmental temperatures. In addition, placental insufficiency during early-mid gestation can be a causative factor for affecting fetal development as formation of primary muscle fibres occurs between d35 and d55 of gestation in fetal pigs^36^. Therefore, the objective of this study was to investigate the impact of controlled elevated temperatures (ET) during early-mid gestation (d40 to d60 of gestation) on placental and fetal development in pregnant pigs.

## Results

### Thermoregulatory responses

**Figure 1:**
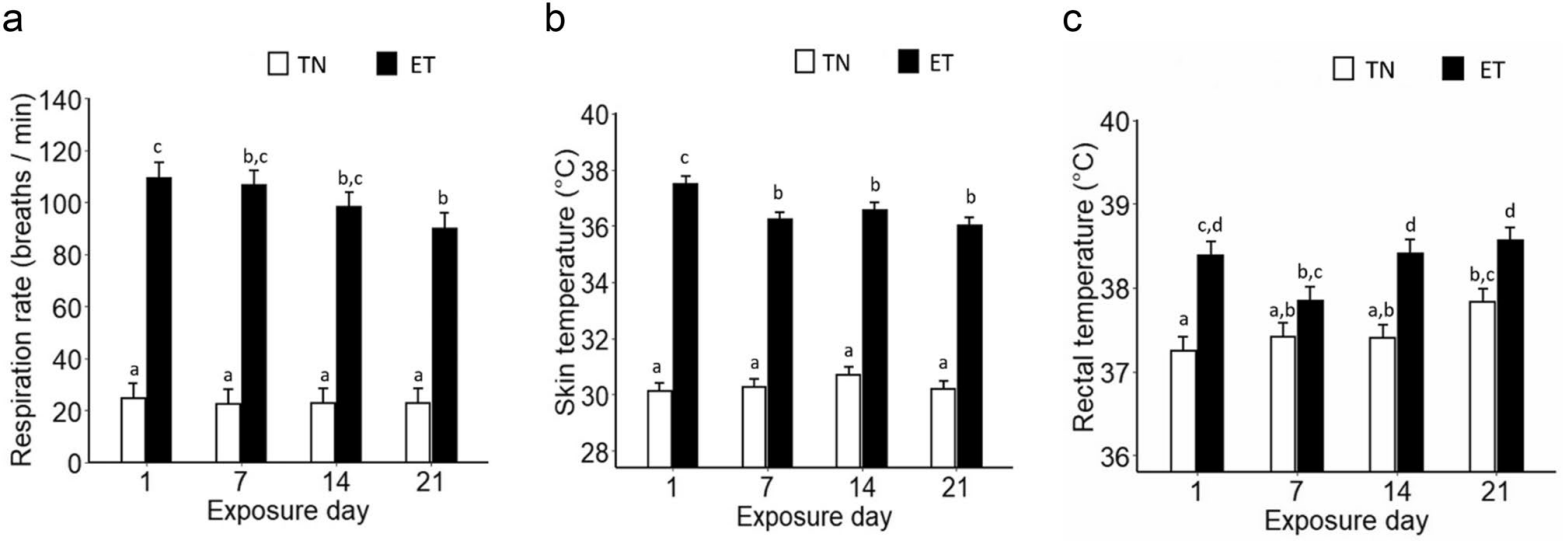
Physiological signs of thermal stress in pregnant pigs exposed to thermoneutral (TN) or elevated temperature (ET) conditions between d40 and 60 of gestation. (**a**) Respiration rate; (**b**) Skin temperature; (**c**) Rectal temperature. TN: n = 7 pigs; ET: n = 8 pigs. Data are expressed as means with average standard error of the difference (SED). Different letters indicate P < 0.05.

On average, ET increased pooled respiration rate of pregnant pigs across the experimental period (23 vs. 101 breaths min^−1^, SED = 6, P < 0.001). There was an interaction between gestational temperature and exposure day (P = 0.017), such that ET pigs had the highest respiration rate on d1, followed by a gradual decrease to the lowest point on d21, while the respiration rate of TN pigs remained constant throughout the experimental period (Fig. 1a).

Overall, pooled skin temperature was increased in ET pigs (30.3 vs. 36.6 °C, SED = 0.3, P < 0.001) throughout the experimental period. A gestational temperature by exposure day interaction was significant (P < 0.001), such that ET pigs exhibited the highest skin temperature on d1 followed by a constantly lower skin temperature over the remainder of the experimental period; whereas TN pigs had a stable skin temperature across the experimental period (Fig. 1b).

Elevated temperatures increased pig pooled rectal temperature by 0.8 °C (37.5 vs. 38.3 °C, SED = 0.1, P < 0.001) as measured across all exposure days. There was an interaction between gestational temperature and exposure day (P = 0.017) as TN pigs had a higher rectal temperature on d21 compared to d1. In contrast, rectal temperatures of ET pigs decreased from initially elevated 38.3 °C at d1 to 37.9 °C at d7 followed by a secondary increase to 38.6 °C until d21 (Fig. 1c).

### Fetal and placental morphometry

There were no significant effects of ET on litter size, total litter weight, within-litter fetal weight variation, uterine weight, ovary weight, fetal survival rate and corpus luteum number (Table 1). Fetal weights were not affected by temperature treatments, but were heavier in males than females, regardless of temperature treatments (P = 0.019; Table 2). Placental weights were increased by 22% (P = 0.041) by ET, but did not differ between females and males (P = 0.61, Table 2). In addition, there was no interaction between fetal sex and gestational temperature for placental weights (P = 0.19). Placental efficiency (fetal/placental weight) was decreased by ET, regardless of fetal sex (P = 0.013, Table 2). Additionally, the interaction between gestational temperature and fetal sex for placental efficiency tended to be significant (P = 0.081), such that female placentae had a greater reduction in placental efficiency by ET compared to their male counterparts. Placentae produced by ET pigs had a larger surface area compared to their TN counterparts (P = 0.049, Table 2). Male fetuses had higher ponderal index (P = 0.007) and body mass index (P = 0.003) than females, regardless of temperature treatments (Table 2). There were interactions between gestational temperature and fetal sex for ponderal index (P = 0.007) and body mass index (P = 0.012), such that the reductions in ponderal and body mass indexes by ET were greater in males than females (Table 2). Similarly, the reduction in head circumference due to ET was greater in males than females (P = 0.002, Table 2) and male fetuses had a greater head circumference compared to female fetuses, regardless of temperature treatments (P = 0.049, Table 2). Neither fetal absolute organ weights, including liver, brain and head, nor their weights relative to fetal weights, were significantly different between fetuses from the two temperature treatments (Table 2).

**Table 1:** Reproductive performance of pregnant pigs exposed to thermoneutral (TN) or elevated temperature (ET) conditions from d40 to d60 of gestation.

Variables	Treatments		SED	P values
	TN (n = 7)	ET (n = 8)		
Litter size	14.1	13.6	1.7	0.73
Within-litter variation of fetal weight (%)	12.8	12.6	1.6	0.88
Total litter weight (g)	1301	1234	140	0.64
Uterine weight (kg)	10.2	10.6	0.9	0.64
Ovary weight (g)	19.0	17.6	2.0	0.52
Corpus luteum number	18.0	16.8	0.7	0.15
Fetal survival rate^a^	0.81	0.71	0.06	0.16

Data are expressed as means with average standard error of the difference (SED).

^a^Fetetal survival rate: number of viable conceptus/number of corpus luteum.

**Table 2:** Fetal and placental morphological variables with pregnant pigs exposed to thermoneutral (TN) or elevated temperature (ET) conditions from d40 to d60 of gestation.

Variables	Temperature treatments									
	TN		ET		TN	ET	SED	P values		
	F (n = 39)	M (n = 38)	F (n = 65)	M (n = 43)				T	S	T * S
**Fetal morphometry**										
Fetal weight (g)	92.9	98.9	90.5	94.5	95.9	92.5	5.4	0.53	**0.019**	0.63
Crown-rump length (CRL, cm)	13.3	12.9	13.7	13.7	13.1	13.7	0.4	0.18	0.25	0.13
Head circumference (cm)	9.8	10.6	9.6	9.5	10.2	9.5	0.6	0.40	**0.049**	**0.002**
**Ponderal index**										
(Fetal weight (g)/CRL (cm)^3^ × 100)	4.10	5.32	3.60	3.71	4.71	3.65	0.78	0.27	**0.007**	**0.007**
**Body mass index**										
(Fetal weight (g)/CRL (cm)^2^ × 100)	53.1	63.7	48.6	50.2	58.4	49.1	7.0	0.29	**0.003**	**0.012**
Placental and fetal organ parameters	n = 12	n = 9	n = 17	n = 7						
Placental weight (g)	85	94	117	103	90	110	13	**0.041**	0.61	0.19
**Placental efficiency**										
(fetal/placental weight)	1.23	1.08	0.85	0.96	1.16	0.91	0.12	**0.013**	0.99	0.081
Placental surface area (cm^2^)	685	702	762	847	693	804	61	**0.049**	0.20	0.42
Head weight (g)	22.1	24.0	21.2	22.0	23.0	21.6	2.1	0.54	0.44	0.70
Brain weight (g)	2.56	2.73	2.57	2.71	2.62	2.64	0.19	0.91	0.08	0.89
Liver weight (g)	5.34	4.66	5.32	5.59	5.00	5.46	0.53	0.45	0.62	0.15
Brain/liver weight	0.518	0.587	0.499	0.486	0.552	0.492	0.055	0.22	0.48	0.29
Head/fetal weight	0.221	0.207	0.221	0.218	0.214	0.220	0.014	0.91	0.49	0.57
Liver/fetal weight	0.055	0.050	0.057	0.057	0.053	0.057	0.004	0.30	0.38	0.35
Brain/fetal weight	0.027	0.029	0.027	0.027	0.028	0.027	0.002	0.52	0.50	0.45

For fetal morphology, including fetal weight, crown-rump length, head circumference, ponderal index and body mass index, fetuses from each pregnant pig were measured (TN: n = 77; ET: n = 108). For placental and fetal organ variables, three focal fetuses from each pregnant pig were measured (TN: n = 21; ET: n = 24). F, Female; M, Male; T, Temperature; S, Sex and T*S, Temperature times sex interaction. Data are expressed as means with average standard error of the difference (SED). Data for the main effect of temperature treatments are pooled means across fetal sex. Significant P values (P < 0.05) are in bold.

### Placental mRNA expression

**Figure 2:**
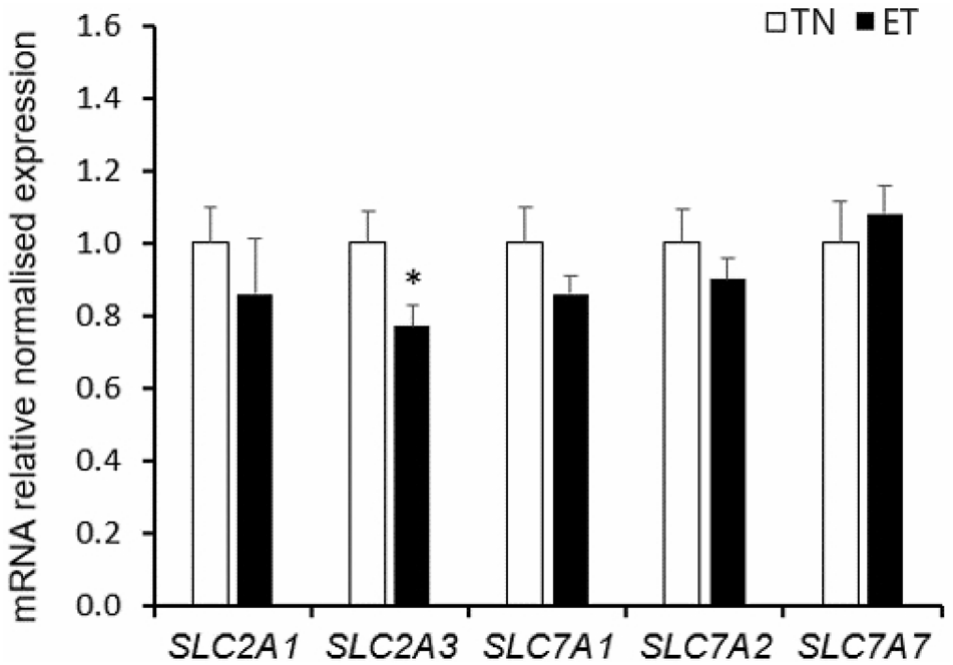
Placental mRNA relative expression of nutrient transporters of pregnant pigs exposed to thermoneutral (TN) or elevated temperature (ET) conditions between d40 to 60 of gestation. *SLC2A1*: solute carrier family 2 member 1; *SLC2A3*: solute carrier family 2 member 3; *SLC7A1*: solute carrier family 7 member 1; *SLC7A2*: solute carrier family 7 member 2; *SLC7A7*: solute carrier family 7 member 7. TN: n = 12 placentae; ET: n = 16 placentae. *P < 0.05.

Placental *IGF-2* mRNA relative expression was increased by ET (1.00 vs. 1.26, SED = 0.12, P = 0.037) while placental *VEGF* mRNA relative expression remained similar between temperature treatments (1.00 vs. 0.95, SED = 0.11, P = 0.29). For placental glucose and amino acid transporters, the mRNA relative expression of *SLC2A3,* a gene encoding glucose transporter GLUT-3, was lower in placentae from ET pigs compared to their TN counterparts (P = 0.026, Fig. 2). Placental mRNA expression of an amino acid transporter, *SLC7A1,* tended to decrease with ET (P = 0.08, Fig. 2). Placental mRNA expression of *SLC2A1*, *SLC7A2* and *SLC7A7* did not differ between ET and TN conditions (Fig. 2).

### Fetal and placental histology

**Figure 3:**
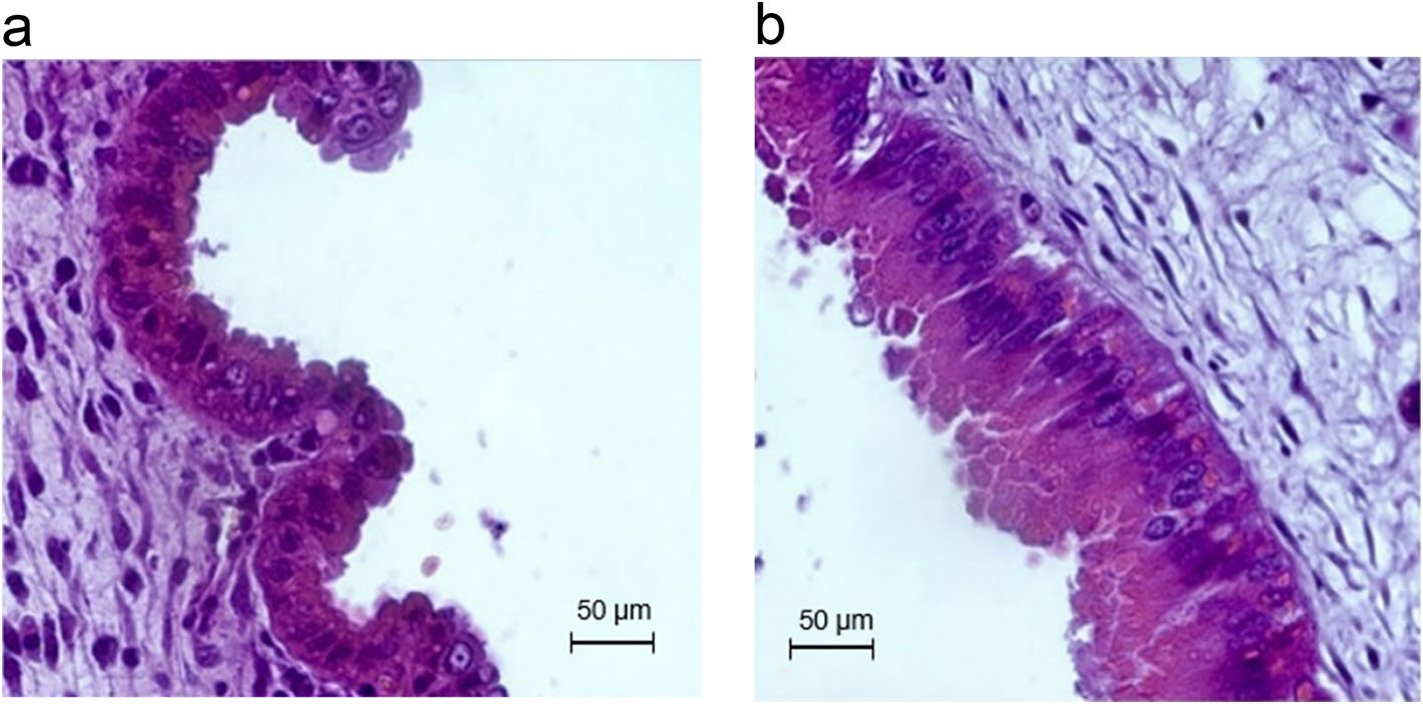
Photomicrographs of representative placental epithelial layer from pregnant pigs exposed to thermoneutral (TN; **a**) or elevated temperature (ET; **b**) conditions. TN: n = 9 placentae; ET: n = 12 placentae.

**Figure 4:**
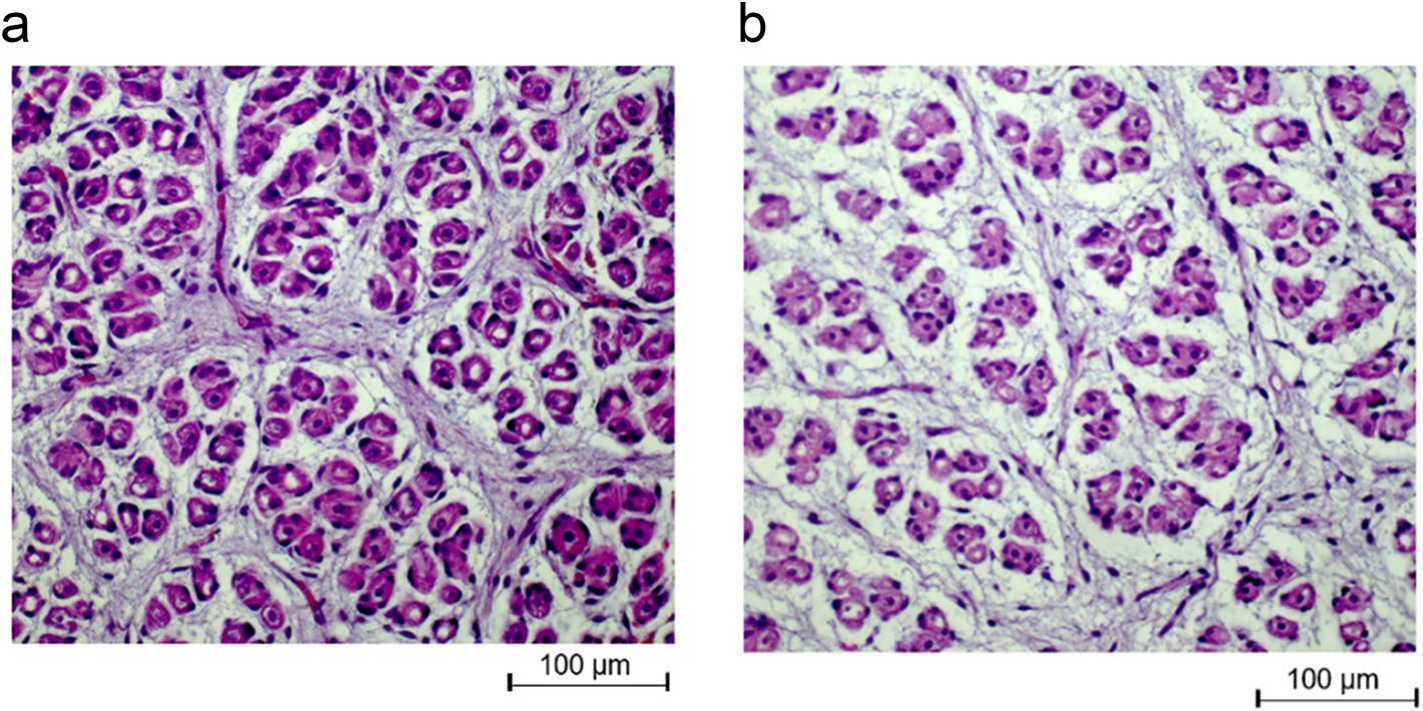
Photomicrographs of representative cross-sectional areas of *M. longissimus* of fetuses from pregnant pigs exposed to thermoneutral (TN; **a**) or elevated temperature (ET; **b**) conditions. TN: n = 14 fetuses; ET: n = 16 fetuses.

Photomicrographs of placental epithelial layers were represented in Fig. 3. Elevated temperatures thickened the placental epithelial layer by 28.6% compared to their TN counterparts (112 vs. 144 µm, SED = 9, P = 0.017). Figure 4 represents a photomicrograph of fetal *M. longissimus*. Compared to fetuses from TN pigs, muscle fibre number density was reduced in fetuses from ET pigs (858 vs. 722 fibres per mm^2^ area of field, SED = 56, P = 0.032). Muscle nuclei number density was lower in fetuses produced from ET pigs than fetuses from TN pigs (1680 vs. 1423 nuclei per mm^2^ area of field, SED = 55, P < 0.001). However, muscle nuclei number per muscle fibre was similar (2.01 vs. 2.02, SED = 0.15, P = 0.96) in fetuses from ET and TN pigs. Moreover, fetal muscle fibre cross-sectional area was not affected by temperature treatments (2.33 vs. 2.40 µm^2^, SED = 0.06, P = 0.51). There were no sex or sex by gestational temperature interactions for muscle fibre parameters.

### Hormone concentrations

Pregnant pigs exposed to ET from d40 to d60 of gestation tended to have lower plasma progesterone concentrations compared to their TN counterparts (17.4 vs. 15.5 ng/mL, SED = 0.9, P = 0.050). Progesterone concentrations per unit mass of placenta were not affected by temperature treatments (162.2 vs. 147.2 ng/mg, SED = 20.1, P = 0.37). Maternal plasma cortisol levels were decreased in ET pigs (6.24 vs. 3.99 µg/dL, SED = 0.91, P = 0.029). Maternal circulating insulin-like growth factor 1 (IGF-1) (81.1 vs. 68.5 ng/mL, SED = 7.9, P = 0.14) and growth hormone (0.78 vs. 1.04 ng/mL SED = 0.21, P = 0.23) concentrations were not significantly different between TN and ET pigs. For maternal plasma thyroid hormones, free T4 concentrations were similar between treatments (0.922 vs. 0.699 ng/dL, SED = 0.164, P = 0.21) but plasma free T3 concentrations were lower (0.360 vs. 0.181 pg/mL, SED = 0.045, P = 0.002) in ET pigs compared to their TN counterparts.

### Venous blood parameters

The effect of ET on maternal auricular ear vein blood parameters are presented in Table 3. Elevated temperatures reduced levels of haematocrit (14%, P = 0.004), haemoglobin (13%, P = 0.009), sodium (4.1%, P = 0.006) and increased blood creatinine levels (27%, P = 0.018, Table 3). Maternal plasma urea nitrogen (PUN) was increased by ET (P = 0.011, Table 3). Other maternal blood variables, such as pH, pCO_2_, pO_2_ and glucose, were similar in pigs between TN and ET treatments (Table 3).

**Table 3:** Auricular venous blood parameters of pregnant pigs exposed to thermoneutral (TN) or elevated temperature (ET) conditions from d40 to d60 of gestation.

Variables	Treatments		SED	P value
	TN (n = 7)	ET (n = 8)		
pH	7.5	7.5	0.0	0.95
pCO_2_ (mmHg)	43.3	42.3	3.1	0.75
pO_2_ (mmHg)	61.3	47.4	8.8	0.14
TCO_2_ (mmol/L)	32.1	31.3	1.4	0.56
Na^+^ (mmol/L)	145	139	2	**0.006**
K^+^ (mmol/L)	4.2	3.9	0.3	0.54
Ca^2+^ (mmol/L)	1.3	1.3	0.1	0.95
CI^-^ (mmol/L)	104	101	2	0.10
Hct (%PCV)	37.3	32.1	1.5	**0.004**
Glu (mmol/L)	5.3	4.9	0.6	0.54
Lac (mmol/L)	2.0	1.3	0.8	0.42
Crea (mg/dL)	2.2	2.8	0.2	**0.018**
PUN (mg/dL)	27.1	48.8	7.2	**0.011**
cHCO_3_^-^ (mmol/L)	30.8	29.9	1.3	0.51
sO_2_ (%)	88.9	80.5	0.1	0.19
cHgb (g/dL)	12.7	11.1	0.5	**0.009**

Data are expressed as means with average standard error of difference (SED). Significant P values (P < 0.05) are in bold.

*pCO*_*2*_ partial pressure of carbon dioxide; *pO*_*2*_ partial pressure of oxygen; *TCO*_*2*_ total carbon dioxide; *Hct* haematocrit; *Glu* glucose; *Lac* lactate; *Crea* creatinine; *PUN* plasma urea nitrogen; *cHCO*_*3*_^−^ bicarbonate; *sO*_*2*_ oxygen saturation; *cHgb* haemoglobin.

## Discussion

The primary findings of the experiment were that exposing pregnant pigs to controlled elevated temperatures (ET) from d40 to d60 of gestation reduces placental efficiency, which would have implications for fetal development. This was evidenced by a reduced fetal to placental weight ratio and a compensatory growth of placentae by ET treatment. The placenta is a key mediator of fetal development by determining the transfer capacity of nutrients and oxygen between maternal and fetal bloodstreams^22^. The insufficiency of placentae produced by ET pigs was also accompanied by reduced maternal progesterone concentrations, possibly affecting pregnancy maintenance^37^. However, in the present study, fetal weights were not significantly impacted by ET. This finding was not surprising because a rapid growth of pig fetuses happens during the second half of gestation^38^. Nevertheless, placental insufficiency during early-mid gestation is likely to constrain fetal development at later stages of gestation when fetal growth rate accelerates. For example, there is compelling evidence that piglet birthweights are reduced by maternal heat exposure during early^17^, late^10^ or the entirety of gestation^39^. Intriguingly, in the current study, a sexually dimorphic effect on placental efficiency in response to ET was observed, such that female placentae tended to be more responsive to ET, compared to male placentae. In fetuses, on the contrary, the impacts of ET were more pronounced in male fetuses, in terms of head circumference, ponderal index and body mass index. This is evidenced by the observations that reductions in those parameters by ET were greater in males compared to females. Ponderal index and body mass index are proxies for evaluating prenatal survivability and development^40^. A decrease in the body metric parameters of those ET male festues may imply that growths of soft tissues or muscle mass may be compromised^32^. These findings are in line with other studies in mammals that male fetuses are more vulnerable to prenatal stress^41,42^.

The novel finding of this study is that pig placentae exhibited adaptations in response to maternal heat exposure. For example, placentae produced by ET pigs were enlarged in terms of total mass, surface area and epithelial layer thickness. In addition, the enlargement of placentae from ET pigs is reflected by increased placental *IGF-2* mRNA expression as *IGF-2* is known to promote placental growth, possibly by enhancing the migration, invasion and differentiation of placental trophoblast cells, and placental nutrient transport^43,44^. Notably, the phenotypic changes in placentae from ET pigs may imply placental compensatory strategies in response to suboptimal in utero conditions^45^. Unlike other mammals, such as humans and rodents, in which the placentae invade into the endometrium and are firmly attached, pigs have non-invasive diffuse placentae^46^. These characteristics suggest additional morphological adaptations, such as an increase in the maternal–fetal interface, may be required to maintain oxygen and nutrient exchange between maternal and fetal circulations^26^. It is speculated that this compensatory enlargement of placental mass and surface area could be more pronounced under suboptimal intrauterine conditions, such as in utero heat stress, where uterine blood flows and nutrient availability are likely to be compromised^30,31^. However, how these morphological adaptations of placentae would help mitigate against the adverse impact of maternal heat exposure, when the demands for fetal growth heavily increase as gestation progresses^38^, remains to be elucidated.

In the present study, placental insufficiency could be explained, at least in part, by decreased placental mRNA expression of *SLC2A3*, a gene encoding a glucose transporter, GLUT-3, although mRNA expression of *SLC2A1*, which encodes for GLUT-1, did not change by ET. Glucose is the primary energy source for fetal development, and the availability of this substrate is heavily determined by placental transport from the maternal circulation^47^. Compared to GLUT-1, GLUT-3 has a higher affinity for glucose and decreased mRNA expression of this gene implies reduced efficiency of placental capacity for glucose transport. In addition to the glucose transporter, ET tended to decrease placental mRNA expression of *SLC7A1*, which encodes a cationic amino acid transporter (CAT-1). In pig placentae, transport of both glucose and amino acids between the maternal–fetal interface is facilitated by the transporters, or carriers, located on the placental membranes^25^. Therefore, decreased gene expression of the transporters suggests compromised placental efficiency. Accordantly, a positive correlation between placental *SLC2A3* mRNA expression and placental efficiency has been demonstrated in late gestating pigs^48^. Although studies investigating the impact of maternal heat exposure on placental nutrient transport in pigs are scarce, the data are supported by previous studies in sheep models, showing that chronic heat stress during gestation reduces glucose transfer capacity in pregnant ewes^30,31^. Similarly, another study^49^ showed that the abundance of placental GLUT-8 mRNA and protein is reduced in pregnant ewes subjected to high temperatures during mid-late gestation. Taken together, the current findings demonstrate that exposing pregnant pigs to ET during early-mid gestation reduced placental gene expression associated with major nutrient transport, possibly impairing placental transport activity. Nevertheless, in the present study, it remains unclear whether decreased mRNA expression of the transporters is a direct consequence of in utero thermal stress or indirectly due to temperature-induced reductions in intrauterine and umbilical blood flows^29,30^.

Another novel finding in the present study is that maternal high temperature exposure during gestation impacts fetal skeletal muscle development as evidenced by that fetuses from ET pigs had fewer muscle fibres in *M. longissimus* compared to the TN fetuses at d60 of gestation. The data accord with the notion that prenatal stress affects muscle fibre hyperplasia of the progeny^50^. In pigs, prenatal muscle fibre hyperplasia is critical for postnatal muscle growth trajectory as muscle fibre number is fixed before birth^36,51^. Reductions in muscle fibre numbers may have implications for decreased capacity for lean tissue accretion and increased adipogenesis later in life^52,53^. Boddicker et al.^20^ demonstrated that grower pigs born from sows that experience heat stress during the first half gestation exhibit elevated thickness of subcutaneous fat with reduced *M. longissimus* cross-sectional area compared with their thermoneutral counterparts, irrespective of postnatal growth conditions. Likewise, Johnson et al.^16^ demonstrated that progeny exposed to prenatal heat stress exhibit higher lipid accretion and lower protein deposition rates at finishing stages compared to the control. In the current study, the mechanisms by which fetuses from pregnant pigs exposed to ET have impaired muscle development are unknown, but are possibly due to compromised myogenesis events, including reductions in myoblast proliferation and expressions of myogenic regulatory factors, as seen in fetuses suffering intrauterine growth retardation (IUGR)^54^. Collectively, the current data, coupled with the literature, suggest that maternal exposure to elevated ambient temperatures during early-mid gestation, when fetal primary muscle fibres heavily develop, compromises fetal muscle fibre growth in pigs, potentially having implications for piglet birthweights and postnatal growth. The impacts might be more drastic when pregnant pigs experience extensive heat events throughout gestation.

## Conclusions

Elevated environmental temperatures during early-mid gestation (d40 to d60) can cause placental insufficiency in pregnant pigs. Although placentae exhibit adaptations as a consequence of maternal heat exposure, those compensatory strategies failed to fully offset the adverse impacts on placentae and fetuses. Placental insufficiency at early gestation may restrain fetal growth potential as gestation progresses, possibly causing a long-term carryover influence on postnatal growth. Further studies investigating interventions to ameliorate the negative impact of thermal stress during gestation on conceptus development are warranted.

## Materials and methods

### Animal ethics

All animal procedures were reviewed and approved by the Animal Ethics Committee of the University of Melbourne, Australia (Ethics Id: 1714365.2). The experimental protocols followed the Australia Code for the Care and Use of Animals for Scientific Purposes^55^.

### Animals and experimental design

Fifteen Large White × Landrace female pigs (gilts) (112 ± 5 kg liveweights, mean ± SD) were selected from a commercial piggery (Huntly Piggery, Victoria) for artificial insemination (AI) over two experimental replicates. Prior to AI, the pigs were oestrus synchronised with an oral altrenogest drench (Regumate, MSD Animal Health, Dublin; 15 mg/day per pig) in water daily for 18 day. Pigs were then artificially inseminated (d0) using the semen (2.0 × 10^9^ viable sperm cells per dose) produced from a single sire on the 4th or 5th day after the last oral altrenogest administration. All mated gilts were group housed in a pen of 8 (1.8 m^2^ floor space per sow, semi-slatted concrete floor) in the semi-climatically controlled gestating shed on the commercial farm (mean maximum and minimum temperature over the early gestation period was 14.5 ± 2.9 °C and 3.4 ± 3.3 °C, respectively, as recoded from the nearest weather station). The pigs were restrictively fed with an average of 2.2 kg diet per day until d28 of gestation. They then had their pregnancies confirmed by ultrasonography before being transported to a climate-controlled facility, University of Melbourne. The pregnant pigs were then individually housed in floor pens (220 cm × 120 cm) in climate-controlled rooms and acclimated to the facility under thermoneutral conditions (TN; constant 20 °C; ~ 50% relative humidity (RH)).

On d40 of gestation, the pregnant pigs were subjected to either constant TN (n = 7) or cyclic elevated temperature conditions (ET, 33 °C and ~ 50% RH between 0900 and 1700 h; 28 °C and ~ 50% RH between 1700 and 0900 h; n = 8) for 3 weeks. All pigs were exposed to an artificial 12 h/day light regimen between 0700 and 1900 h. Overall, the temperature treatment duration was between d40 and d60 of gestation. Throughout the experiment the pregnant pigs were limit-fed with a commercial sow gestation diet twice daily at 2.0 kg a day, which contained 13.0 MJ/kg digestible energy (DE) and 0.6% lysine^56^. Therefore, the nutrition intake was similar for pigs between each temperature treatment. All pigs had ad libitum access to water.

### Physiological signs of thermal stress

Physiological signs of thermal stress of pregnant pigs were assessed by weekly (on day 1, 7, 14, 21) recordings of skin temperature and respiration rate at 0900, 1100, 1300, 1500 and 1700 h. Rectal temperature was measured weekly at 0900 and 1500 h. Respiration rate was assessed by visually counting flank movements using a stopwatch and expressed as breaths per minute. Rectal temperature was assessed using a digital thermometer (Surgipack; Vega Technologies Inc., Dongguan, China). Skin temperature of the flank sites was assessed by a non-invasive laser thermometer (Digitech Inc., Zurich, Swiss). One decimal place can be read from the thermometer and the temperatures were measured using the same thermometer for all pigs in each treatment in order to minimise the error.

### Blood sampling and euthanasia

On the final day of the treatments, pregnant pigs were sedated with intramuscular injections of Xylazil-20 and Ketamine (Troy Laboratories Pty Ltd, NSW, Australia) at 0.6 mg/kg and 10 mg/kg liveweight, respectively. Ten mL of maternal blood was collected by jugular venepuncture into sodium heparin vacutainers (BD, North Ryde, NSW, Australia) and the blood was immediately centrifuged at 2000 × *g* for 10 min at 4 °C for plasma collection. The plasma samples were then aliquoted and stored at -20 °C prior to further analysis. In addition, 1 mL blood was collected via a needle and syringe from the auricular vein in pregnant pigs and immediately loaded into an automatic blood gas analyser (Epoc; Alere, Waltham, MA, USA) for venous blood analysis. Pregnant pigs were then euthanised via intracardiac injections of Lethabarb (pentobarbitone sodium; 162.5 mg/kg liveweight; Virbac Animal Health, NSW, Australia).

### Tissue collection and morphometric analysis

In each pregnant pig, the gravid uterus was harvested via mid-ventral laparotomy and the total weight was recorded. Then, the uterus was opened along the antimesometrical side, and the fetuses were numbered, their exact location in the tract recorded before they were carefully separated from the endometrium. For each uterus, one side of the uterine horn was randomly chosen, from which three focal fetuses from the tip, middle and cervical locations were identified. For focal fetus (n = 45), fetal head, brain, liver and *M. longissimus* were dissected and weighted. Three matched focal placentae from each litter were peeled from the endometrium. The ovaries were dissected and the number of corpus luteum was counted. Litter size, fetal weight, placental weight, fetal sex, crown-rump length (CRL), head circumference, placental efficiency (fetal/placental weight), ponderal index (fetal weight (g)/CRL (cm)^3^ × 100), body mass index (fetal weight (g)/CRL (cm)^2^ × 100), within-litter fetal weight variation and fetal survival rate (number of viable conceptus/number of corpus luteum) were recorded and calculated. Placental surface area was measured by using an A3 cutting mat with grids (1 cm × 1 cm) and calculated by counting the total number of the grid covered. One section of placental tissue near the umbilical cord area from each focal fetus was harvested, snap frozen in liquid nitrogen and stored at − 80 °C prior to further biochemical and molecular assays. A subset sections of placentae (TN: n = 9; ET: n = 12) and fetal *M. longissimus* (TN: n = 14; ET: n = 16) were harvested and fixed with 4% paraformaldehyde in 0.1 M phosphate-buffered saline (PBS; pH 7.4) prior to histological processing.

### Hormone assays

Plasma progesterone, thyroid (free thyroxine (T4) and triiodothyronine (T3)) and cortisol concentrations were measured using radioimmunoassay (RIA) kits as per the manufacturer’s instructions (MP Biomedicals, Orangeburg, USA), with specific radioactivity measured with a gamma counter (PerkinElmer Life Science, Massachusetts, USA). The average of intra-assay CVs for progesterone, cortisol and free T4 and T3 were 1.7%, 3.5%, 4.7% and 3.7%, respectively. Total insulin-like growth factor 1 (IGF-1) concentrations were quantified using a commercial ELISA kit (R&D systems Inc., Minneapolis, USA) following the manufacturer’s instructions. The intra-assay CV and minimum detectable dose were 9.3% and 0.026 ng/mL, respectively. Plasma growth hormone (GH) concentrations were measured using a commercial pig ELISA kit (USCN, China) according to the manufacturer’s protocols. The sensitivity of this assay was 0.117 ng/mL and the intra-assay CV was 3.9%. Placental tissue progesterone concentrations were measured using a competitive ELISA kit (Cayman Chemical Co., Ann Arbor, MI, USA) according to the manufacturer’s instructions. The intra-assay CV and assay sensitivity were 5.2% and 10 pg/mL, respectively.

### Placental RNA isolation, cDNA synthesis and real-time polymerase chain reaction (qPCR)

Placental total RNA was extracted using the column based ReliaPrep RNA tissue Miniprep System (Promega, Madison, WI, USA) as per the manufacturer’s instructions. Twenty grams of starting tissues were used for RNA extraction. RNA integrity was determined by using an automatic microfluidic gel electrophoresis system and the Experion RNA StdSens Analysis kit (Bio-Rad Laboratories Inc., Hercules, Ca, USA) according to the manufacturer’s instructions. RNA purity was assessed using a nanodrop spectrophotometer (Thermo Scientific, Walton, MA, USA). A total of 28 RNA samples (TN: n = 12; ET: n = 16) with qualified RNA quality (RNA quality indicator (RQI) ≥ 7 and A260/A280 between 1.8 to 2.2) were chosen for downstream qPCR experiments. A total of 1 µg RNA was used for the synthesis of first-strand complementary DNA (cDNA) using the GoTaq 2-Step qPCR System (Promega, Madison, WI, USA) as per the manufacturer’s instructions. Real-time PCR reactions were performed using SYBR Green I and the PCR results were presented and analysed using the gene study model within CFX Manager Software (Bio-Rad Laboratories Inc., Hercules, Ca). The reactions were performed in a final volume of 10 µL containing 5 µL of GoTaq qPCR Master Mix, 0.5 µL of each forward and reverse primer, 1 µL of cDNA template and 3 µL of nuclease-free water. Two references genes, including ribosomal protein L32 (*RPL32*) and eukaryotic translation elongation factor 1 alpha 1 (*EEF1A1*), were chosen as they exhibited acceptable stability among treatments (M-value = 0.27 and 0.19 for *RPL32* and *EEF1A1*, respectively). Primer sequences of reference and target genes that regulate placental development, including insulin-like growth factor 2 (*IGF-2*), vascular endothelium growth factor (*VEGF*)*,* and placental transporters of glucose (*SLC2A1* and *SLC2A3*) and amino acid (*SLC7A1, SLC7A2, SLC7A7*), were obtained from the National Center for Biotechnology Information (NCBI) gene database and listed in Supplementary Table S1 online. The primers were designed using PrimerQuest Tool^57^ with specificity against the porcine genome being checked using the NCBI Nucleotide Blast program. The amplification efficiency of each target gene was evaluated. The thermal cycling conditions were programmed by the following steps: (1) DNA polymerase activation for 2 min at 95 °C for 1 cycle; (2) DNA denaturation for 15 s at 95 °C; (3) Primer annealing for 45 s at 55 °C followed by an extension for 15 s at 72 °C. The denaturation, annealing and extension steps were then repeated for a total of 40 cycles. Each sample was run in triplicate. The Cq values of genes of interest were normalised to the reference genes using the 2^−ΔΔCq^ method^58^.

### Muscle and placental tissue microscopy

The fixed fetal *M. longissimus* samples (TN: n = 14; ET: n = 16) were processed as described by Gatford et al.^59^. The cross-sectional area of the tissues was processed and embedded in cassettes filled with paraffin wax. Then, serial transverse sections were cut at a thickness of 8 µm by a rotary microtome (Leica biosystems, Victoria, Australia). Two serial sections were picked onto a glass microscope slide followed by staining with haematoxylin and eosin (H&E). Tissue sections were then imaged using a microscope (Zeiss, Oberkochen, Germany) under a magnification of × 20 objective. For each section, five fields were randomly chosen and imaged, and a total of ten fields were captured. Muscle fibre number, nuclei number and muscle cross-sectional area were quantified using the ImageJ software (NIH, USA). Data were presented as muscle fibre number density (muscle fibre number per mm^2^ field). Muscle nuclei abundance was analysed with the threshold and particle analysis function within the ImageJ software and presented as muscle nuclei number density (nuclei number per mm^2^ field) and nuclei number per muscle fibre.

The placental tissues (TN: n = 9; ET: n = 12) were transversely embedded in cassettes filled with paraffin wax. The paraffin-embedded tissue blocks were sectioned at a thickness of 6 µm by a rotary microtome. Two serial sections from each block were picked onto a glass microscope slide followed by the H&E staining. Placental epithelial layers were chosen as the focus of morphometric measurements as described by Vallet et al.^26^. Briefly, a microscopic field with epithelial layer area was chosen, from which lines were drawn to connect the bottom and top of the layer. The average width of the placental epithelial length was calculated as the average length of the lines. Five microscopic fields were chosen per each section, and a total of ten fields were measured per placenta sample.

### Statistical analysis

Data were verified a normal distribution and analysed by liner mixed models (REML) (Genstat 18th ed, VSN International, Hemel Hempstead, UK). For maternal parameters, including reproductive traits, auricular vein blood parameters and hormone concentrations, data were analysed for the main effect of gestational temperature with experimental replicate being a random factor. For sequential measures of pregnant pig thermoregulatory responses, including respiration rate, rectal and skin temperatures, data were analysed with fixed factors being gestational temperature, exposure day and all appropriate interactions. Random factors for the model were dam and experimental replicate. The data for the main effect of gestational temperature are presented as pooled means across all exposure days. For fetal and placental measures, gestational temperature, fetal sex and all interactions were designed as fixed factors with dam (nested within gestational treatment) being the random effect. The data for the main effect of gestational temperature or fetal sex are presented as pooled means. The effect of fetus position relative to uterine horn was removed from the model as there was no impact on any parameters. For placental gene expression analysis, the main effect of gestational temperature was included in the statistical model. The data are expressed as relative normalised expression with TN treatment being set as the control. All data are presented as means with average standard error of the difference (SED). A statistical significance was considered when P values were < 0.05 and a trend was considered when 0.05 ≤ P ≤ 0.1.

## Supplementary information

Supplementary Information.

## Data Availability

The datasets generated during and/or analysed during the current study are available from the corresponding author on reasonable request.
