# Supplementary material for: Controlled elevated temperatures during early-mid gestation cause placental insufficiency and implications for fetal growth in pregnant pigs
Source: Sci Rep. 2020 Nov 26;10:20677. doi: 10.1038/s41598-020-77647-1 (PMC7691357; doi:10.1038/s41598-020-77647-1)
Supplement: Supplementary file 1 — Supplementary Information. [file 41598_2020_77647_MOESM1_ESM.pdf]

Supplementary information

Controlled elevated temperatures during early-mid gestation cause placental insufficiency and implications for fetal growth in pregnant pigs

Weicheng Zhao, Fan Liu, Alan W. Bell, Hieu H. Le, Jeremy J. Cottrell, Brian J. Leury, Mark P. Green, Frank R. Dunshea

Supplementary Table S1 List of sequences of primers used for real-time polymerase chain reaction (qPCR)

Gene symbol	Encoding proteins	Primer sequences (5' → 3')	Accession number
IGF-2	Insulin-like growth factor-2	Forward – ATT GGC TTG GTC TGT ATC TTC<br>Reverse – GAT GGT ATG TGG GTG TGG TT	NM_213883.2
VEGF	Vascular endothelial growth factor	Forward – TAT GCG GAT CAA ACC TCA CC<br>Reverse – CTT GCC TCG CTC TAT CTT TCT T	NM_214084.1
SLC2A1	Facilitated glucose transporter-1 (GLUT-1)	Forward – GGC ATG TGC TTC CAG TAT GT<br>Reverse – GTC TCG GGA ACT TTG AAG TAG G	X17058.1
SLC2A3	Facilitated glucose transporter-3 (GLUT-3)	Forward – CGA TCG TCA TCG GGA TTC T<br>Reverse – ATG ATG GTG AAG CCC AAG AG	AF054836
SLC7A1	Cationic amino acid transporter-1 (CAT-1)	Forward – CCA TGC CGC GAG TTA TCT AT<br>Reverse – GAG GCT AAC GTG GCG ATT AT	NM_001012613.1
SLC7A2	Cationic amino acid transporter-2 (CAT-2)	Forward – GGC AGC TTT GAC CCT TAT GA<br>Reverse – AAC GAC ATA CTT GGC AGG AC	NM_001110420.1
SLC7A7	Y+L amino acid transporter 1 (y+LAT1)	Forward – GCC TCG ACT CAC TTT GAG AA<br>Reverse – AGC CCG AGT AAG AGA AGA GA	NM_001110421
RPL32	Ribosomal protein L32	Forward – AGG GTC ACC AAT CCC AAT G<br>Reverse – GGA TAC TAG CTG GGT GCT TAA T	NM_001001636
EEF1A1	Eukaryotic translation elongation factor 1 alpha 1	Forward – CTG AAC GTG AGC GTG GTA TTA<br>Reverse – CTG TGT CCT GGA GCA TCA AT	NM_001097418
